# Biotechnological Potential and Safety Evaluation of Dextran- and Riboflavin-Producing *Weisella cibaria* Strains for Gluten-Free Baking

**DOI:** 10.3390/foods13010069

**Published:** 2023-12-24

**Authors:** Pasquale Russo, Iñaki Diez-Ozaeta, Nicola Mangieri, Mercedes Tamame, Giuseppe Spano, Maria Teresa Dueñas, Paloma López, Mari Luz Mohedano

**Affiliations:** 1 Department of Food Environmental and Nutritional Sciences (DeFENS), University of Milan, 20133 Milan, Italy; pasquale.russo1@unimi.it (P.R.); nicola.mangieri@unimi.it (N.M.); 2 Departamento de Biotecnología Microbiana y de Plantas, Centro de Investigaciones Biológicas Margarita Salas (CSIC), 28040 Madrid, Spain; i.diez08@hotmail.com (I.D.-O.); plg@cib.csic.es (P.L.); 3 Departamento de Química Aplicada, Facultad de Química, Universidad del País Vasco (UPV/EHU), 20018 San Sebastián, Spain; mariateresa.duenas@ehu.eus; 4 Instituto de Biología Funcional y Genómica (IBFG), CSIC-Universidad de Salamanca, 37007 Salamanca, Spain; tamame@usal.es; 5 DAFNE Department, University of Foggia, 71122 Foggia, Italy; giuseppe.spano@unifg.it

**Keywords:** *Weissella cibaria*, riboflavin, dextrans, gluten-free, functional bread, clean label

## Abstract

Gluten consumption causes several immunological and non-immunological intolerances in susceptible individuals. In this study, the dextran-producing *Weissella cibaria* BAL3C-5 and its derivative, the riboflavin-overproducing strain BAL3C-5 C120T, together with a commercial bakery yeast, were used to ferment gluten-free (GF)-doughs obtained from corn and rice flours at two different concentrations and supplemented with either quinoa, buckwheat, or chickpea to obtain laboratory-scale GF bread. The levels of dextran, riboflavin, and total flavins were determined in the fermented and breads. Both strains grew in fermented doughs and contributed dextran, especially to those made with corn plus quinoa (~1 g/100 g). The highest riboflavin (350–150 µg/100 g) and total flavin (2.3–1.75 mg/100 g) levels were observed with BAL3C-5 C120T, though some differences were detected between the various doughs or breads, suggesting an impact of the type of flour used. The safety assessment confirmed the lack of pathogenic factors in the bacterial strains, such as hemolysin and gelatinase activity, as well as the genetic determinants for biogenic amine production. Some intrinsic resistance to antibiotics, including vancomycin and kanamycin, was found. These results indicated the microbiological safety of both *W. cibaria* strains and indicated their potential application in baking to produce GF bread.

## 1. Introduction

Gluten consumption may cause several gluten-related disorders (GRDs), including coeliac disease, dermatitis herpetiformis, gluten ataxia, and non-coeliac gluten sensitivity (NCGS) [1]. In recent years, the market for gluten-free (GF) products has been growing worldwide, and their sales are expected to reach 6.47 billion USD by 2023, with an annual growth rate of 7.6% [2]. Gluten-replacing alternatives include GF flour from cereals (i.e., millet, sorghum, teff, maize and rice), pseudocereals (i.e., quinoa, amaranth, and buckwheat), and legumes (i.e., chickpea, soy, and carob germ) [3,4]. Although GF bakery products can be made using different GF flours to improve their nutritional value, their use may cause some critical technological issues [5,6]. Indeed, in breadmaking, gluten acts as a structural network contributing to viscoelasticity after the hydration of flour, resulting in an improved crumbling texture in the bread and improved color and sensory qualities after baking [7]. Replacing gluten functionality is still a challenge for breadmaking and often requires the addition of strengthening additives such as gums and hydrocolloids, emulsifiers, non-gluten proteins from milk, eggs, legumes and pulses, enzymes, and non-starch polysaccharides [7,8]. Currently, consumers increasingly tend to consume products made using as many natural and healthy ingredients as possible, avoiding the consumption of foods containing synthetic ingredients. The addition of pseudocereal and legume flours improves the nutritional and structural characteristics of bakery products. These flours act as water binders and film formers, eliminating the need for other additives [9].

Sourdough is a complex ecosystem mainly characterized by the leavening action of yeasts and lactic acid bacteria (LAB), with a prevalence of heterofermentative lactobacilli [10]. It is well-known that some LAB species and/or particular LAB strains are able to produce exopolysaccharides (EPS), high molecular weight polymers that display physicochemical properties similar to commercial hydrocolloids [11]. In particular, LAB strains of species belonging to the genus *Weissella* are good producers of dextrans and sometimes are found as inhabitants of spontaneously fermented (Type 1) traditional sourdough [12,13,14]. Thus, *Weissella* spp. strains have been proposed as inocula to improve the viscoelasticity of the final GF bakery dough, to enhance the volume softness of the bread crumb and textural properties of the loaves, to provide an anti-staling effect, to retard starch retrogradation, and to prolong the shelf life of bread [15,16,17]. Therefore, in situ dextran production is considered an interesting opportunity for the production of GF bakery goods [18,19,20]. Accordingly, the employment of dextran-producing *Weissella cibaria* strains significantly improved the overall quality of sourdough obtained from buckwheat [21,22,23], chickpea [24], and quinoa flours [16,25].

Furthermore, dextrans synthesized by *W. cibaria* have been reported to be bioactive compounds with immuno-stimulant and anti-inflammatory properties [25], suggesting their possible ability to improve the functionality of bakery products. In recent studies, *W. cibaria* strains isolated from a rye sourdough made in Spain and the corresponding roseoflavin-resistant riboflavin-overproducing derivatives have been employed to biofortify in situ with riboflavin and dextran wheat laboratory bread [26,27,28]. Riboflavin (vitamin B_2_) is an essential compound mainly involved in the respiratory chain, which exerts antioxidant effects and is synthesized by food-grade micro-organisms [29]. Thus, riboflavin biofortification of fermented foods using spontaneously overproducing strains belonging to other LAB species has been reported as a valuable biotechnological approach to enhance the nutritional quality production of laboratory bread [30,31] and functional fermented beverages [32,33,34]. However, riboflavin biosynthesis during fermentation is strongly affected by the food matrix, therefore requiring a careful selection of microbial sources for specific productions [33].

Recently, clean-label foods obtained with minimal additives have gained popularity in the food market and are considered an attractive alternative that induces positive attitudes in consumers [35,36]. Thus, the employment of LAB strains that are able to synthesize in situ dextrans and high levels of riboflavin during fermentation offers interesting applications in the food industry both from a technological and functional point of view.

In this study, the dextran-producing *W. cibaria* BAL3C-5 and its derivative, the strain BAL3C-5 C120T (to our knowledge, this is the described *W. cibaria* strain producing the highest riboflavin levels) [28], have been used as coadjuvant together with a commercial bakery yeast to ferment pilot scale GF dough obtained by mixing different combinations of corn or rice GF flour with ones from pseudocereals or legumes. The riboflavin and dextran production has been determined in laboratory-scale bread. The main safety features of both *W. cibaria* strains have also been evaluated using in silico and in vitro approaches. The overall results indicate that *W. cibaria* BAL3C-5 C120T is suitable for the safe biofortification of GF bread with riboflavin and dextran.

## 2. Materials and Methods

### 2.1. Microbial Strains and Growth Conditions

*Weissella cibaria* BAL3C-5 previously isolated from rye sourdough [37] and its roseoflavin-resistant riboflavin-overproducing *W. cibaria* BAL3C-5 C120T [28] were used in this work. LAB strains were grown at 30 °C in De Man–Rogosa–Sharpe (MRS) broth (Oxoid, Basingstoke, UK). The commercial strain *Saccharomyces cerevisiae* Lievital (Lesaffre Italia, Parma, Italy) was isolated on plates of yeast extract peptone dextrose (YEPD) and routinely grown in YEPD broth (Oxoid) at 28 °C with shaking (75 rpm).

### 2.2. Breadmaking Assays at Laboratory Scale

#### 2.2.1. Preparation of the Dough

Flours of corn, rice, quinoa, buckwheat, and chickpea were provided by Molendum Ingredients S.L. (P.I. El Roto c/ Milana, 49530, Coreses, Zamora, Spain). Flours (75 g) were obtained by adding corn and rice flours at two different concentrations, namely 60–20% or 40–40%. The remaining 20% contained flour made from quinoa, buckwheat, or chickpea. The use of pseudocereals and legume flours was based on previous studies (reviewed in [10]). The choice of high concentration of the legume flour was based on previous work [38]. Distilled water containing 3% sucrose and 1.5% NaCl was added to the flour (1:1 *v*/*w*) and manually kneaded. The obtained dough was aliquoted into three portions, each of 50 g.

#### 2.2.2. Inoculum of the Dough

Doughs were fermented with *S. cerevisiae* Lievital cultured in the laboratory (control breads) or in combination with either *W. cibaria* BAL3C-5 or BAL3C-5 C120T strains. Microbial biomass was recovered by centrifugation (5000× *g*, 3 min) and washed twice with sterile saline solution. The pellet was resuspended in the same initial volume. A microbial suspension (500 µL) was added to the center of the ~50 g dough samples to obtain an expected final concentration of approximately 1.0 × 10^7^ colony-forming units (CFU)/g for bacteria and 1.0 × 10^6^ CFU/g for the yeast. In control samples, 500 µL of sterile saline solution were added to obtain the same final volume. The dough was manually kneaded to homogenize the microbial suspension and further divided into three portions of 15 g each. Fermentation was carried out at 30 °C for 16 h using a nonstick 24-cup mini-muffin pan. The choice of the conditions for inoculation and fermentation was based on our previous research on the preparation of experimental breads with *W. cibaria* strains [27] and another LAB [30,31]. Then, one sample was used to determine the microbial load, while two replicates were baked at 210 °C for 15 min. Samples were covered with aluminum foil to avoid loss of photosensitive riboflavin and stored at −20 °C until further analysis.

### 2.3. Microbiological Analysis

Microbiological analyses were performed on the microbial suspensions used to inoculate the dough and on one fermented sample for each experimental condition. Viable cells of yeast and bacteria were enumerated after growing on plates of YEPD supplemented with chloramphenicol (10 µg/mL) or MRS supplemented with cycloheximide (10 µg/mL) at 30 °C for 48 h.

### 2.4. Dextran Extraction and Quantification

The dextran synthesized by the LAB strains could be in a soluble or insoluble form. Therefore, soluble and total dextrans were independently extracted and quantified as previously reported [27]. Briefly, to extract soluble dextran, samples of flour, dough, and bread (0.75 g) were suspended in distilled water (1.5 mL) and incubated at 20 °C for 24 h, with shaking at 200 rpm. Then, they were treated with *Chaetomium erraticum* dextranase (Sigma-Aldrich, Darmstadt, Germany, 180 mg in 150 μL) for 24 h at 30 °C, with shaking at 200 rpm, to convert dextran into isomaltose. Afterward, samples were centrifuged at 8000× *g* for 10 min, and supernatants were filtered using a 0.22 µm filter and stored at −20 °C until further analysis. To extract total dextran, flour, dough, and bread samples (1 g) were resuspended in 0.1 M HCl (10 mL), autoclaved at 121 °C for 30 min, and the pH of the samples increased to 6.5 by addition of sodium acetate pH 8.0, prior to conversion of the polymer into isomaltose by dextranase treatment as described above.

Experiments were performed in duplicate, and the dextran concentration was determined after hydrolysis by quantification of the generated isomaltose by gas chromatography-mass spectroscopy (GC-MS) using myo-inositol as an internal standard as previously described [27].

### 2.5. Riboflavin Extraction and Quantification

The flour may contain, in addition to free riboflavin and flavins synthesized by the micro-organisms, other flavins that will also be present in the dough and the bread. Therefore, we extracted not only water-soluble riboflavin but also any other flavins present that were converted into riboflavin prior to quantification. Riboflavin and flavins were extracted as previously reported [27] with slight modifications. Briefly, for riboflavin testing, samples of bread (0.75 g) were suspended in 1.5 mL of ultrapure water and vigorously vortexed for 10 min. After incubation for 24 h at 20 °C with shaking at 200 rpm, they were treated with *C. erraticum* dextranase (as in 2.3) for 24 h at 30 °C. Then, samples were centrifuged (20 min, 13,400× *g*). The supernatants were filtered using a 0.22 μm filter. Flour and dough samples were obtained using the same procedure: 0.375 g of individual or mixed flours according to each experimental condition supplemented with 0.375 g of distilled water containing 3% sucrose and 1.5% NaCl.

For total flavin extraction and conversion into riboflavin, flour, dough, and bread samples were processed for total dextran extraction and treatment with dextranase, as in 2.4. Then, samples were centrifuged (20 min, 13,400× *g*), and the supernatants were filtered using 0.22 μm filters.

Riboflavin present in all filtered samples was quantified by fluorescence spectroscopy with excitation at 440 nm and detection of emission at 520 nm, using a Varioskan Flask System (Thermo Fisher Scientific, Waltham, MA, USA). The concentration of the riboflavin was determined using calibration curves, as previously described [39].

### 2.6. Safety Evaluation

#### 2.6.1. Antibiotic Resistance

The total cell count and viability of the bacterial cultures were assessed by flow cytometry, following the ISO 10932 procedure [40], with some modifications as previously reported [41]. Bacterial cultures were grown at 30 °C for 24 h in ISO-Sensitest broth (Oxoid) supplemented with 10% *v*/*v* MRS (Oxoid) and diluted in filtered phosphate-buffered saline (PBS, pH = 7.4) to achieve a concentration of approximately 1.0 × 10^6^ cells/mL. An events rate in the flow lower than 2000 events s^−1^ was maintained. The samples were stained with 0.1 µM SYTO™ 24 (Thermo Fisher Scientific) and 0.2 µM propidium iodide (PI; Sigma) and incubated in the dark at 37 °C for 15 min. Flow cytometry analysis was carried out with a C6 Plus flow cytometer (BD Biosciences, Milan, Italy) with thresholds FSC-H 1000 and SSC-H 1000. All parameters were collected as logarithmic signals. Green (SYTO™ 24) and red (PI) fluorescence were detected in the FL1 (excitation 488 nm, emission filter 530/30) and FL3 (excitation 488 nm, emission filter 670 LP) channels, respectively. Electronic gates on the SYTO24/PI density plot were used to select and measure the total bacterial concentration (events mL^−1^), active fluorescent unit (AFU), and non-active fluorescent unit (nAFu), as described in ISO 19344 (2015) [42].

According to the EFSA food additives panel (EFSA-FEEDAP) document 2012, the antibiotics included in the analysis were those tested for Gram-positive bacteria: ampicillin, vancomycin, gentamicin, kanamycin, streptomycin, erythromycin, clindamycin, tetracycline, and chloramphenicol (Merck, Darmstadt, Germany). Minimal inhibitory concentrations (MIC) for the antimicrobials listed above were determined by the broth dilution method in 96-well plates, using 10 antimicrobial concentrations (128; 64; 32; 16; 8; 4; 2; 1; 0.5 and 0.25 μg/mL) with a bacterial inoculum density of 5 × 10^5^ AFU/mL. To standardize the bacterial density in the inoculum suspensions, cell counting was performed by flow cytometry as described above. The bacterial cultures containing 5.75 × 10^8^ AFU/mL for the wild type and 5.62 × 10^8^ AFU/mL for the mutant were diluted to a final concentration of 1 × 10^6^ AFU/mL in the appropriate medium, and 0.1 mL of this dilution was added as inoculum in a final volume of 0.2 mL. Controls were inoculated into broth without antimicrobials. The plates were incubated at 30 °C for 24 h. The experiment was conducted in duplicate. The minimum inhibitory concentration MIC was the lowest concentration of antimicrobial, where no visible growth was measured in the wells.

#### 2.6.2. Hemolysin Activity

Hemolysin activity was evaluated through zigzag streaking of frozen cultures of the *W. cibaria* strains and *Streptococcus pneumoniae* JNR7/87 as α-hemolytic control on Columbia agar plates containing 5% horse blood (Oxoid) and then incubated at 30 °C for 48 h. A clear zone of hydrolysis around the colonies (β-hemolysis), partial hydrolysis with green coloration (α-hemolysis), or no hydrolysis around colonies (γ-hemolysis) were evaluated. Only γ-hemolysis is considered safe, as previously described [43].

#### 2.6.3. Gelatinase Activity

Gelatinase activity was tested on nutrient agar containing 30 g/L gelatin, 5 g/L peptone, 3 g/L yeast extract, and 15 g/L agar. The overnight cultures of the *W. cibaria* strains were spot-inoculated and incubated at 30 °C for 48 h. After incubation, saturated ammonium sulfate was added to plates, and the presence of a clear zone around the colonies was considered a positive result of gelatinase activity, as previously described [44].

### 2.7. Genome Analysis: Safety Evaluation

The genome sequence was then further evaluated using BLAST software for comparison and detection of specific genetic traits. Antibiotic resistance and virulence and pathogenicity factors were evaluated by genome comparison against CARD (https://card.mcmaster.ca/) (accessed on 13 March 2023) [45], ResFinder (ResFinder 4.1 (dtu.dk) (accessed on 13 March 2023) [46], IslandViewer4 (Islandviewer 4—Genomic Island Prediction and Genome Visualization Tool (sfu.ca)) and VFDB (Virulence Factor Database; VFDB: Virulence Factor Database (mgc.ac.cn) (accessed on 13 March 2023) [47] databases, respectively. Finally, CRISPR and prophage elements were also analyzed through CRISPRFinder [48] and PHASTER [49] databases, respectively. The resulting intact prophages were submitted to the Virus-Host DB database [50], and a similarity tree of the viral genome sequence was generated with the VIPtree tool [51]. Prediction of the biogenic amines (BA) synthesis ability of BAL3C-5 strains was performed through BLASTp software by alignment of reference genes related to histamine, tyramine, putrescine, and cadaverine production. Since no strain belonging to *W. cibaria* species has been described and annotated in databases as BA producer to date, reference genes of LAB species were considered for comparison purposes.

### 2.8. Statistical Analysis

The experiments, including analysis of dextran and flavin production, were analyzed with a one-way ANOVA. A *p*-value of ≤ 0.05 was considered significant. When ANOVA tests were significant, mean pairwise comparisons were computed with a Tukey’s test (α = 0.05), and results are shown with letters; means with the same letter are not significantly different. All analyses were performed with the R software version 4.3.0 [52].

## 3. Results and Discussion

### 3.1. Safety Evaluation of the W. cibaria Strains

*W. cibaria* BAL3C-5 C120 is a riboflavin-overproducing strain selected by roseoflavin treatment of the dextran-producing BAL3C-5 strain isolated from a rye sourdough [37]. The selected BAL3C-5 C120T constitutive mutant is able to produce high concentrations of dextran and riboflavin in growth medium [28]. Therefore, it could be a good candidate for the development of functional GF bread. However, although different *Weissella* strains have been investigated for their probiotic and biotechnological potential, the genus *Weissella* is not included in the list of qualified presumption of safety (QPS) biological agents by the European Food Safety Administration (EFSA) [53]. Indeed, while *Weissella* spp. has been found during the fermentation of different matrices, some strains have been reported to be involved in disease outbreaks [54,55]. In fact, this genus is usually categorized as an opportunistic pathogen, mainly due to *W. confusa*-linked illnesses in immunodeficient individuals [56,57]. However, in recent years, there have been multiple examples of the health-promoting characteristics of potential probiotic *Weissella* spp. strains, mainly belonging to *W. cibaria* species, have been evaluated. Among these, the immunomodulatory, anticholesterolemic, antioxidant, antimicrobial, and potential antiviral and anticancer properties of different *Weissella* spp. have been confirmed. [58,59,60,61,62]. Nevertheless, a preliminary strain safety assessment of *Weissella* strains is required for application in the food industry [63]. Consequently, prior to testing *W. cibaria* BAL3C-5 C120T and its parental BAL3C-5 strain for their performance fermenting pilot GF dough, they were subjected to safety evaluation. The entire genome of both *W. cibaria* strains was previously determined, and they only differ in one nucleotide (C120T) responsible for constitutive riboflavin production [28]. Thus, in silico analysis of the genomes was performed to determine if the genetic determinants of other hazard factors were present.

#### 3.1.1. In Silico Analysis of Genetic Determinants for Biogenic Amine Biosynthesis

The first aspect considered was to predict the ability of these strains to produce biogenic amines (BA), which are low molecular weight nitrogenous compounds that can be found naturally or through microbial activity in various foods. Spoiled foods and fermented foods are common matrices containing variable concentrations of BA, and one of the main groups responsible for their production is LAB [64]. The most common BA found in food matrices are histamine, tyramine, putrescine, and cadaverine [65]. Although it was thought that only histamine and tyramine represented a toxicological risk, recently, it has also described the cytotoxic effect of both putrescine and cadaverine [65]. Prediction of the BA synthesis ability of BAL3C-5 and BAL3C-5 C120T strains was performed with BLASTp software by alignment of reference genes encoding the biosynthetic pathways of histamine, tyramine, putrescine, and cadaverine. No positive result was recorded for any of the BA analyzed. Therefore, the use of these strains in food fermentations should not lead to BA production.

#### 3.1.2. In Vivo and In Silico Analysis of Antibiotic Sensitivity

In some cases, LAB are not recognized as GRAS micro-organisms, partly due to an antibiotic resistance profile or the presence of pathogenicity factors. Thus, the resistance of the two *W. cibaria* strains was tested following the instructions of the EFSA for the screening of bacterial products intended for use as feed additives [66]. Although in recent years, an increasing number of studies have addressed the safety of *Weissella* spp. strains, MIC breakpoints have not yet been defined by EFSA, making it complex to define sensitivity or resistance to clinical antibiotics within this genus.

Indeed, a lack of homogeneity in the reference epidemiological cutoff values for *Weissella* spp. hinders the interpretation of the results. For example, Fanelli et al. [63] considered that strains are resistant to a specific antimicrobial when the MIC was >10-fold the cutoff value described by the EFSA-FEEDAP for other Gram-positive bacteria. In contrast, Fhoula [67] used the cutoff values adopted for Leuconostoc spp. as a reference [66] and those reported for *Weissella spp.* by Suhonen [68], while Quattrini et al. [69] compared the values reported for *Lactobacillus* and *Leuconostoc* spp. Moreover, other studies refer only to the EFSA cutoff values of *Lactobacillus* obligate heterofermentative species [59,70]. However, according to recent relevant scientific literature [67,69,71,72] and in an attempt to provide a standardized interpretation guideline, in this work, we have determined the antibiotic susceptibility based on the EFSA cutoff value of *Leuconostoc* spp., which reflects the phylogenetic proximity to the *Weissella* genus.

Table 1 shows the phenotypic susceptibility of both strains, and the obtained values of the MIC were compared to the epidemiological cutoff values based on the recommendation of EFSA for *Leuconostoc* spp. [66], since for *Weissella* spp., there are currently no EFSA cutoff values.

Vancomycin resistance was detected for both strains, and this trait was previously reported within the *Weissella* genus [55,63,67,73,74]. Like many LAB, this resistance may be considered intrinsic and not transmissible since *Weissella* spp. possesses, in its cell wall, a peptidoglycan ending in D-Ala-D-Lactate instead of the D-Ala-D-Ala required for high-affinity binding of the antibiotic [69,75]. The MIC breakpoints for kanamycin were marginally higher than those for *Leuconostoc*, as previously reported [72]. Accordingly, higher breakpoint values for this aminoglycoside (64 mg/L instead of 16 mg/L) have been recently suggested [69]. Indeed, *W. cibaria* strains isolated from Kimchi possessed a MIC ≥ 256 mg/L for kanamycin, suggesting that the resistance against this antibiotic could be considered an intrinsic property [71].

Interestingly, BAL3C-5 and BAL3C-5 C120T were resistant to tetracycline. In general, *Weissella* spp. are susceptible to this antibiotic [55,70,74], while only a few strains have been reported as resistant to 30 mg/L [67,76]. However, screening of genes involved in tetracycline resistance has been found in different *Weissella* spp. [63]. In the case of the BAL3C-5 and BAL3C-5 C120T, the gene involved in the tetracycline resistance was not located in a mobile element and presumably not transmissible. This assumption is based on the fact that, according to a tblasttn analysis performed here, the transposable *tetM* and the plasmid-carried *tetL* genes are not present in their bacterial genomes, nor in the other 13 *W. cibaria* genomes sequenced until now.

Furthermore, besides the tetracycline and the non-transmissible vancomycin resistances, our results support the susceptibility of the two *Weissella* strains to the evaluated antibiotics since they showed MIC values lower than the cutoff values established by the EFSA for food-grade micro-organisms. Moreover, after analyzing and comparing the genome of BAL3C-5 strains with the sequences available in CARD and ResFinder databases, no resistance genes were observed.

#### 3.1.3. In Vivo and In Silico Analyses of Virulence Factors

Moreover, when genome analysis was evaluated in search of virulence factors associated with genes, no positive result was obtained through the Islandviewer4 and VFDB databases. In this sense, no result was found for curated virulence factors, homologs or virulence factors, curated resistance genes, homologs of resistance genes, and pathogen-associated genes.

**Figure 1:**
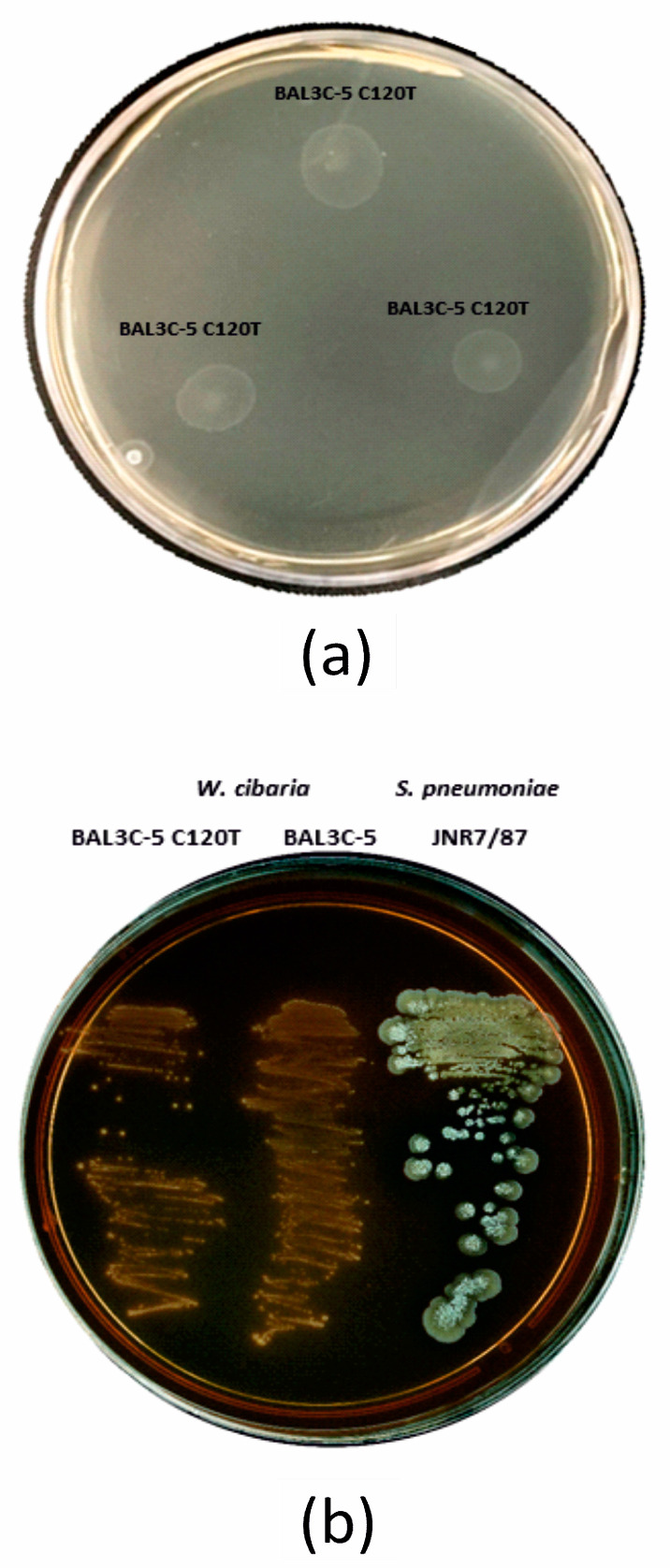
Phenotypical assay for gelatinase and hemolytic activities. (**a**) Evaluation of three spots of *Weissella cibaria* BAL3C-5 C120T, showing no gelatinase activity. (**b**) Evaluation of *W. cibaria* BAL3C-5 C120T and BAL3C-5, showing non-hemolytic activity as well as *Streptococcus pmeumoniae* JNR7/87 with α hemolytic activity.

Moreover, since β-hemolysis activity is related to pathogenicity and gelatinase activity is known as a pro-inflammatory factor, the two strains were tested for these activities, and no hemolytic or gelatinase activities were detected (Figure 1). None of the genetic determinants of these features, together with cytolysin, hyaluronidase, C3-degrading protease, C5a peptidase, IgA protease, exotoxins, serine protease, and neuraminidase activities, which are considered potential virulence factors, were found after the genome analysis. The absence of these activities is considered an essential characteristic for the selection and potential utilization of these strains in the food industry [44,77,78,79].

Thus, considering the above characterization, *W. cibaria* BAL3C-5 C120T does not appear to contain genes, giving rise to safety concerns, and its potential could be investigated as a coadjuvant for functional food development.

### 3.2. Elaboration of GF Laboratory Bread

#### 3.2.1. Selection of Flour for Preparation of the Dough

The flour, which is more often used to prepare GF bakery products with corn or rice, does not provide the best rheological properties to the bread, and in general, its content of vitamin B_2_ is usually low.

Therefore, in this study, we investigated the inoculation of *W. cibaria* BAL3C-5 and BAL3C-5 C120T to ferment GF corn and rice pilot dough supplemented with flour of pseudocereals or legumes, with the aim to develop new types of biofortified and functional bread.

Flour milled from rice (*Oryza sativa*) and corn (*Zea mays*) is the most commonly used for GF breadmaking due to its properties including, among others, being hypoallergenic as well as containing a high concentration of digestible carbohydrates and low levels of sodium chloride [80,81]. However, although these gluten-free mixtures contain a high carbohydrate content, they are deficient in proteins, and excessive consumption may result in a nutritional deficiency in essential components such as vitamin D and B, iron, zinc, calcium, magnesium, and fiber [82]. Therefore, the addition of other components to the mixtures containing proteins is advisable [80]. Thus, to enhance the nutritional value of the bread, we have tested the incorporation of three different flours: quinoa, buckwheat, and chickpea. Quinoa flour contains high levels of lysine, methionine, cysteine, calcium, iron, and phosphorus and is a good source of vitamins E and D [83]. Buckwheat flour has phagopyritols, a type of soluble carbohydrate that enhances glycemic control in patients with insulin-dependent diabetes. This disease is highly correlated with coeliac disease. Buckwheat flour has a low glycemic index, which contributes to the regulation of blood pressure and cholesterol metabolism [84]. Buckwheat- and quinoa-containing bread have a greater volume than other varieties of GF bread [85]. Chickpea flour is a rich source of calcium, magnesium, zinc, potassium, and phosphorus and possesses iron levels similar to most GF flour but with a lower sodium content [86]. Therefore, these three selected flour could be substituted with wheat flour to produce highly nutritious GF bread [87]. Moreover, we utilized a high proportion of the three flours (20%) to achieve an optimal enrichment, as previously described [38].

Thus, in addition to flour milled from corn and rice, flour from a legume (chickpea, *Cicer arietinum L*.), and two pseudocereals quinoa (*Chenopodium quinoa*) and buckwheat (*Fagopyrum esculentum*) rich in proteins and some functional metabolites (i.e., folate and polyunsaturated fatty acids) were chosen as special flour to be used in this work.

#### 3.2.2. Analysis of the Dextran and Flavin Content of the Selected Flour

**Figure 2:**
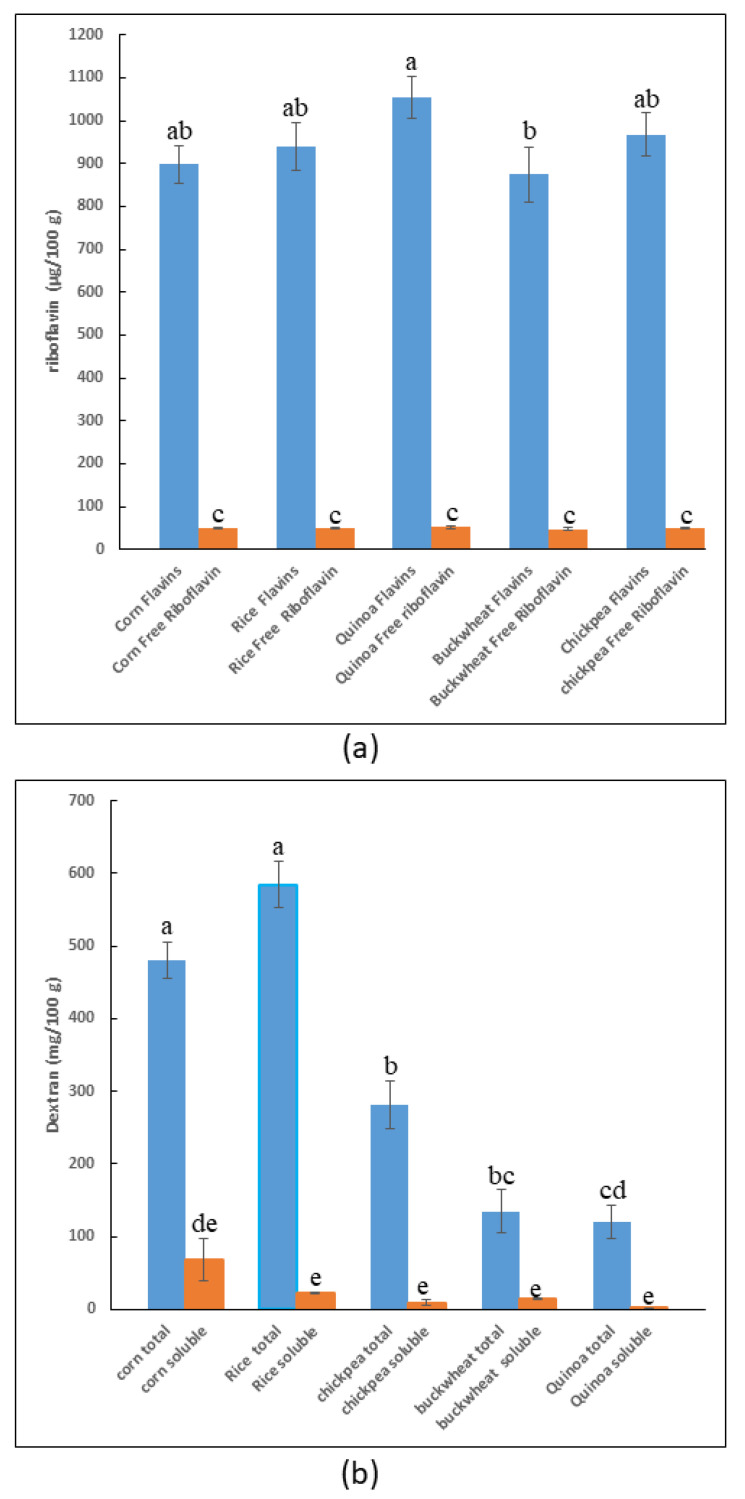
Analysis of flour. Concentration of total flavins and riboflavin (**a**) as well as of total and soluble dextrans (**b**) in the corn, rice, chickpea, and buckwheat flour is depicted. The letters indicate different statistical groups inferred from the Tukey’s test (α = 0.05).

The selected types of flour were analyzed for their content of riboflavin and total flavins after conversion in the former by measuring its fluorescence (Figure 2a). As expected, the levels of water-soluble (free) riboflavin in the flour were low and similar (37 ± 3 μg/100 g). Furthermore, the total flavin concentration was much higher than the former (947 ± 70 μg/100 g), a difference with statistical significance.

Concerning the dextran, we have previously shown that the polymer content in sorghum could be determined by quantification of the isomaltose generated by treatment with *Chaetomium erraticum* dextranase [27]. Thus, the same method was used to measure the total and soluble dextran present in the flour (Figure 2b). Variable levels were observed depending on the flour matrix and whether it was water-soluble or insoluble. For total dextran, the levels ranged from 584 to 120 mg/100 g in rice and quinoa, and for soluble dextran, from 69 to 0.82 mg/100 g, with the highest values for corn and quinoa flour. In addition, corn flour contained a high concentration (482 mg/100 g) of total dextran. Nevertheless, an increase in dextran concentration in GF bread could improve the organoleptic properties of these bakery products.

#### 3.2.3. Experimental Design for Production of Laboratory Bread

**Figure 3:**
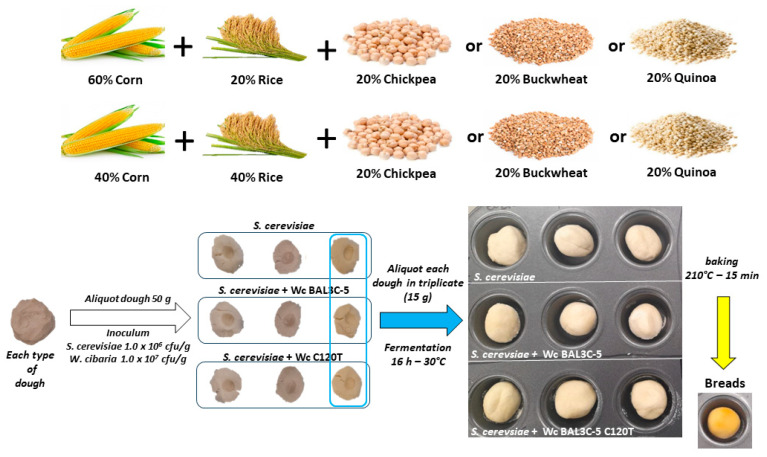
Schematic representation of the workflow for making GF laboratory bread. The combination of flours used is indicated in the upper part of the figure. The steps from dough-making until the production of the baked bread are indicated in the lower part of the figure.

On these bases, we decided on the following flour combinations and experimental design for testing the *W. cibaria* BAL3C-5 C120T in comparison with its parental BAL3C-5 strain (Figure 3 and see details in Section 2.2). The previously tested types of flour were mixed to different extents. In particular, mixtures containing 80% corn and rice flour at two different weight ratios (40%:40% and 60%:20%) were used due to the high content of dextran of those two flours. In addition, these combinations of corn and rice were supplemented with 20% chickpea, quinoa or buckwheat, with the aim of increasing protein, folate, and fatty acid concentration, not provided by rice and corn, and decreasing the carbohydrate concentration provided by them.

#### 3.2.4. Analysis of *W. cibaria* and Yeast Survival after Fermentation

In recent years, the adaptability of LAB and yeasts to ferment flour matrices from different matrices, including special grains, pseudocereals, and legumes, has been widely reported [88]. Therefore, yeast (the commercial *Saccharomyces cerevisiae* Lievital) and either *W. cibaria* BAL3C-5 C120T or BAL3C-5 strains were inoculated in the pilot dough at a cell concentration (determined by plate counting) of 7.5 × 10^5^ CFU/g and 3.0 × 10^7^ CFU/g, respectively. This value is consistent with the microbial composition of some traditional sourdough, characterized by a concentration of LAB of at least 1 log higher than yeasts, and thus would favor the imposition in the pilot dough of the selected micro-organisms over the indigenous flour microbiota. In addition, as a control, each type of dough was inoculated with only yeast. All types of flour matrices supported the growth of the inoculated micro-organisms well, and the corresponding cell counts are summarized in Table 2.

Indeed, after 16 h of fermentation when inoculated alone, *S. cerevisiae* achieved a final concentration 1–2 log higher, increasing from the inoculated 7.5 × 10^6^ CFU/g to 8.1 × 10^7^–2 × 10^8^ CFU/g in all dough samples, indicative of the growth and fermentative performance of the yeast regardless of the flour matrices. In contrast, in mixed fermentation with *W. cibaria,* the viability of the yeast was similar or sometimes slightly lower, a result likely attributable to some competition dynamics between the two inoculated microbial populations. This observation is not so obviously explained since different competition or positive mutualistic interactions between LAB and yeasts in sourdough fermentation have been reported [89]. However, in dough containing buckwheat flour, the viability of *S. cerevisiae* further decreased approximately to 3 × 10^7^ CFU/g and 6 × 10^7^ CFU/g, suggesting some effect of the matrix under mixed fermentation conditions of the yeast with the LAB strains. Accordingly, a study investigating the adaptability of LAB and yeasts to different pseudocereal sourdough yeasts could not be detected in buckwheat sourdough, probably due to the high level of rutin and like-tannin compounds [88]. The final levels of both *Weissella* strains after a 16 h fermentation were always higher than 1 × 10^9^ CFU/g, except in samples containing quinoa where concentrations were even 1 log lower (Table 2), confirming that the molecular composition of the flour has different effects on the microbial strain and/or species growth used as inocula for fermentation.

### 3.3. Biofortification of the GF Bread

**Figure 4:**
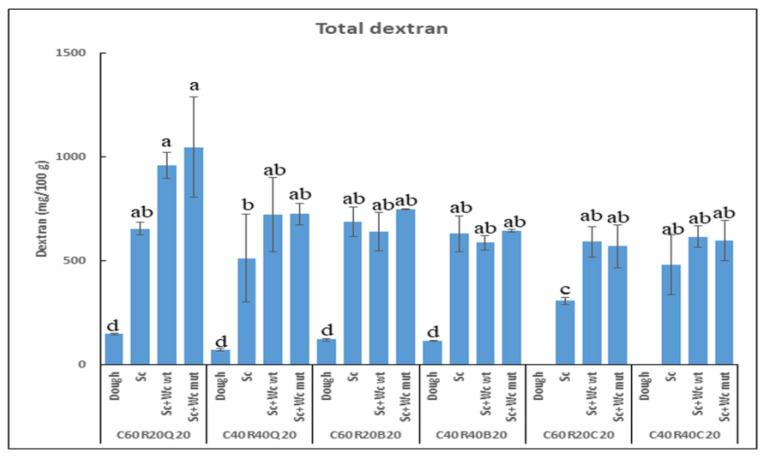
Evaluation of total dextran in pilot unfermented dough and laboratory bread. The levels of the polymer were evaluated in doughs (first bar on the left) and baked breads prepared with 60% or 40% of corn (C60 or C40), 20% or 40% of rice (R20 or R40), and 20% quinoa (Q20) or 20% buckwheat (B20) or 20% chickpea (C20) and fermented only with yeast (control, Sc), or co-fermented with yeast and either *W. cibaria* BAL3C-5 or *W. cibaria* BAL3C-5 C120T (Sc+Wc wt or Sc+Wc mut). No dextran was detected in dough containing chickpea (the detection limit was 4 mg/100 g). The letters indicate different statistical groups inferred from the Tukey’s test (α = 0.05).

**Figure 5:**
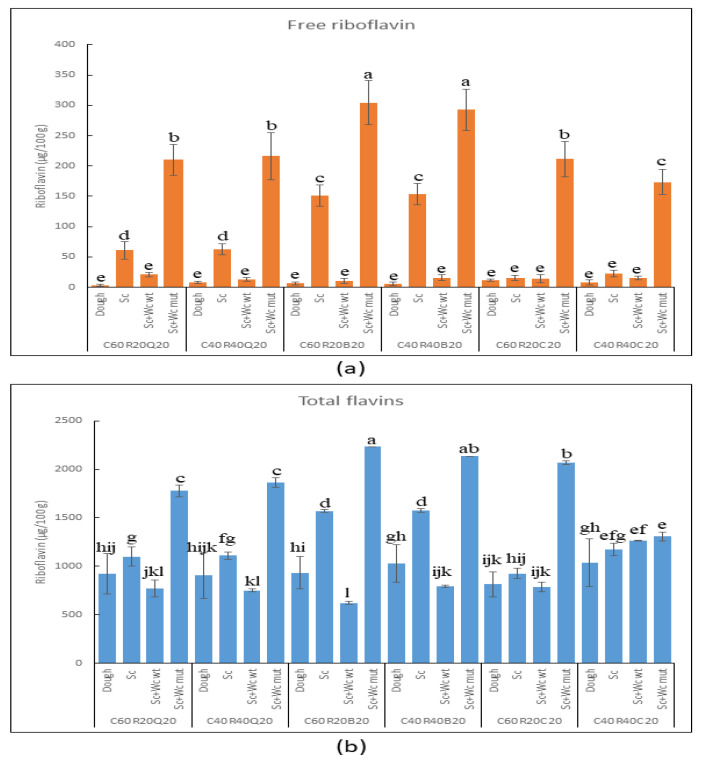
Evaluation of flavins in pilot unfermented dough and laboratory bread. Free riboflavin (**a**) and total flavin (**b**) contents in doughs and breads prepared with 60% or 40% of corn (C60 or C40), 20% or 40% of rice (R20 or R40), and 20% quinoa (Q20) or 20% buckwheat (B20) or 20% chickpea (C20). The letters indicate different statistical groups inferred from the Tukey’s test (α = 0.05).

After the micro-organism fermentation for 16 h at 30 °C, the laboratory bread was obtained by baking the dough at 210 °C for 15 min (Figure 3), and the contents of dextran (Figure 4) as well as riboflavin and total flavin (Figure 5) were determined.

#### 3.3.1. Detection of Total Dextran in Pilot Dough and Laboratory-Produced Bread

Levels of soluble dextran below the detection levels were present in the dough and the bread (results not shown). The analysis of total dextran present in the bread revealed that fermentation of the different doughs resulted in a statistically significant increase of dextran concentration, the highest levels being those obtained with matrices containing quinoa (around 1 g/100 g) co-fermented with yeast and either of the two *Weissella* strains. In the case of samples containing quinoa and those containing chickpeas and 60% corn, the co-fermentation of the yeast with either LAB resulted in a statistically significant increase of dextran production (around 1.5-fold) in comparison with the control (only fermented by yeast) levels. LAB are the more frequent producers of different dextrans; however, some *S. cerevisiae* strains also produce this type of polymer [90].

In this context, the results presented suggest that the *S. cerevisiae* Lievital strain used here can produce dextran and that the co-metabolism of this yeast with the *W. cibaria* strains should be synergistic in some cases and at least useful for the development of some functional GF bread biofortified with dextrans.

Finally, the above results indicate that the co-metabolism of the commercial *S. cerevisiae* Lievital with the *W. cibaria* BAL3C-5 or *W. cibaria* BAL3C-5 C120T strains should be useful for the development of functional GF bread biofortified with high molecular weight dextran. Moreover, these findings further support the employment of dextran-producing *Weissella* strains to counteract the quality deficiencies introduced by gluten network disruption and to balance the negative effects of wheat-flour substitution with legume flour [91,92].

#### 3.3.2. Detection of Riboflavin and Total Flavins in Pilot Dough and Laboratory Bread

Concerning the production of riboflavin (Figure 5a), non-fermented dough contained only a minimal amount of the free vitamin B_2_ (7 ± 3 μg/100 g), the highest level being detected in bread containing 60% corn, 20% rice, and 20% chickpea (12 μg/100 g).

In general, pseudocereal and legume flour are poor sources of vitamin B_2_ because they are often (not always) submitted to the physical removal of some grain fractions, as well to processes responsible for the loss of thermolabile, water-soluble and photosensitive compounds [93,94,95]. In general, no significant differences were detected among bread made with the two different ratios of corn and rice. Interestingly, control samples fermented only with the yeast showed different levels of riboflavin, indicating a significant role of the *S. cerevisiae* strain in producing this vitamin that seems to be matrix-dependent. Indeed, while in bread containing chickpea fermented only by the yeast, no increase of riboflavin was detected, a concentration of about 62 µg/100 g was quantified in bread containing quinoa, while a further increase up to a concentration of 150 µg/100 g was found in buckwheat samples. In recent years, *S. cerevisiae* has been found to generally increase the folate content during sourdough fermentation [96,97]. Less known is the contribution of bakery yeast in riboflavin production, although stepwise fermentation (total of 6 h) of whole wheat using only *S. cerevisiae* as a starter resulted in a 30% enrichment in vitamin B_2_ [98]. However, our results indicated a different ability to synthesize riboflavin by the commercial yeast strain, probably modulated by the flour, since no differences in the viability of *S. cerevisiae* were found in dough fermented only with this micro-organism. Also, it has been reported that environmental stress conditions such as nutritional and oxidative stress induced riboflavin overexpression in *Ashbya gossypii* [99], while iron-limiting conditions encouraged overproduction of riboflavin in a variety of yeasts that are typical inhabitants of sourdough such as *Candida* spp. and *Pichia guilliermondii* [100]. However, these hypotheses are only speculative, and further investigation should be addressed to determine if a reduced micronutrient availability could occur in buckwheat, encouraging riboflavin production.

Interestingly, a strong reduction (around 12-fold) in the level of riboflavin with respect to the control (only fermented by yeast) was detected in buckwheat samples in mixed fermentation with *W. cibaria* BAL3C-5 and to a lower extent (around 4-fold) in quinoa samples. These results could be partially explained by a lower growth of *S. cerevisiae* in dough containing buckwheat, as well as by the consumption of the riboflavin available in the food environment by *W. cibaria* BAL3C-5 strain. This detrimental effect on the vitamin content was observed during fermentation of rye sourdough, where yeasts increased the folate contents while selected LAB strains remarkably decreased its concentration [101]. Similarly, a reduction of about 60% and 36% of riboflavin has been reported when the riboflavin-producing strains *Lactiplantibacillus plantarum* UFG8 and *Limosilactobacillus fermentum* PBCC11 were co-cultured with Caco-2 cell lines, respectively [102]. In contrast to riboflavin-producing strains, the selection of roseoflavin-resistant *W*. *cibaria* mutant strains (including BAL3C-5 C120T) exhibited a constitutive expression of the *rib* operon regardless of the occurrence of exogenous riboflavin [27,103]. Accordingly, higher levels of riboflavin have always been observed in this work in mixed fermentation of the commercial yeast with the *W. cibaria* BAL3C-5 C120T strain. In particular, the matrix containing buckwheat flour achieved a vitamin B_2_ concentration of 300 µg/100 g, while no statistical differences were detected among bread containing chickpea and quinoa flour (approximately 200 µg/100 g). However, considering the contribution of the bacteria (vitamin B_2_ production in mixed fermentation versus that in yeast fermentation), chickpea-supplemented flour was the matrix supporting the highest riboflavin production, which increased between 14- and 8-fold, whereas, in the presence of quinoa or buckwheat, the increase was of 4- or 2-fold, respectively. Noteworthy, the detected level of riboflavin was enough to meet more than 20% of the RDA for adult individuals, considering an average daily intake of about 4 slices of bread (25–30 g each slice) [104]. In a recent study, different *W. cibaria* overproducing strains employed to ferment white wheat flour resulted in bread containing around 125 µg/100 g of free riboflavin [27]. Although the vitamin B_2_ concentration in laboratory dough and bread was lower than that found in the present study, it must be considered that fermentation in that study was performed without yeast and that BAL3C-5 C120T is a very high, constitutive riboflavin-overproducing strain, as determined in culture media [28]. In a pioneer work on bread biofortification, a riboflavin-overproducing *L. fermentum* strain was able to increase the B_2_ content to 666 μg/100 g [31]. However, it is worth underlining that this amount includes the total flavins extracted by submitting the sample food to acidic and enzymatic hydrolysis. Thus, when the total flavin concentration present in the bread was determined (Figure 5b), much higher levels were observed in the co-fermentation of the yeast with *W. cibaria* BAL3C-5 C120T. In all the matrices tested, the flavins concentration in bread was higher than 1750 μg/100 g, reaching values of 2300 μg/100 g in the flour buckwheat-containing samples, proving a better behavior of this *W. cibaria* riboflavin-overproducer than that of the *L. fermentum* strain previously tested [31]. However, it should be stated that in all matrices, the increase over the corresponding control sample (dough inoculated only with commercial yeast) was only 2-fold. Moreover, slight differences were observed between the flavin levels detected in the dough and in the control samples, with the highest increase (around 1.6-fold) in the case of the fermented buckwheat flour-containing samples. Finally, in this analysis was also detected a decrease of flavin concentration in samples co-fermented with the *W. cibaria* BAL3C-5 strain compared with their controls, being the highest decrease that observed in the buckwheat-containing samples.

Thus, the overall results support the constitutive production of riboflavin by *W. cibaria* BAL3C-5 C120T in GF pilot dough and the enrichment by this strain in the content of total flavins in 15 min baked experimental bread. Finally, it should be stated that the determination of so-called free riboflavin (i.e., the water-soluble fraction), performed here, should be a better estimation of the bioavailable and bio-accessible vitamin B_2_ after bread ingestion, and it is worthwhile to perform this type of analysis.

## 4. Conclusions

In this study, we showed that the *W cibaria* BAL3C-5 C120T mutant could be considered to be a potential inoculum for some applications in the bakery product industry. The intrinsic ability of this species to produce dextrans makes these underexploited LABs of great interest in producing some types of GF bread. Moreover, our data indicate that the employment of this selected riboflavin-overproducing and dextran-producing BAL3C-5 C120T strain is a consolidated approach to elaborate some in situ biofortified bakery goods. Therefore, these results suggest biotechnological solutions, both from a technological and functional point of view, compatible with the clean-label concept. In addition, it is expected that the increase of dextran content in the GF bread would improve these bakery products’ rheological properties. However, to prove this assumption, the organoleptic properties of the bread must be tested. Also, the functionality of the bread enriched in dextran and riboflavin should be tested in the future in animal models prior to evaluation in human trials.

Finally, the safety of both strains confirms their deliberate introduction into the food chain, providing supplementary information for regulatory agencies, such as EFSA, in the framework of a comprehensive assessment of the QPS status of species belonging to the *Weissella* genus.

## Figures and Tables

**Table 1:** Determination of minimum inhibitory concentration (mg/L) of the indicated antibiotics for *W. cibaria* BAL 3C-5 and BAL3C-5 C120T strains.

Bacteria	Ampicillin	Vancomycin	Gentamicin	Kanamycin	Streptomycin	Erythromycin	Clindamycin	Tetracycline	Chloramphenicol
*Leuconostoc* spp.	**2**	**n.r.**	**16**	**16**	**64**	**1**	**1**	**8**	**4**
BAL3C-5	1	>128	4	32	32	<0.25	<0.25	32	4
BAL3C-5 C120T	1	>128	2	32	16	<0.25	<0.25	32	4

The MIC were assessed using cutoff concentrations as described by EFSA. Epidemiological cutoff values for *Leuconostoc* spp. are in bold. Each assay was performed in duplicate. n.r. not required.

**Table 2:** Micro-organism viability in the pilot dough after 16 h fermentation.

Flour		Inocula	*S. cerevisiae* (CFU/g)	*W. cibaria* (CFU/g)	Flour	*S. cerevisiae* (CFU/g)	*W. cibaria* (CFU/g)
Corn (40%)—Rice (40%)	Chickpea (20%)	Sc	2.09 × 10^8^	<1.00 × 10^4^ LAB *	Corn (60%)—Rice (20%)	1.89 × 10^8^	<1.00 × 10^4^ LAB
		Sc+Wc wt	7.60 × 10^8^	1.80 × 10^9^		7.80 × 10^7^	1.90 × 10^9^
		Sc+Wc mut	7.70 × 10^7^	1.58 × 10^9^		6.10 × 10^7^	1.40 × 10^9^
	Quinoa (20%)	Sc	1.38 × 10^8^	<1.00 × 10^4^ LAB		1.11 × 10^8^	<1.00 × 10^4^ LAB
		Sc+Wc wt	1.02 × 10^8^	1.03 × 10^9^		9.00 × 10^7^	5.80 × 10^8^
		Sc+Wc mut	6.80 × 10^7^	1.90 × 10^8^		7.80 × 10^7^	2.5 × 10^8^
	Buckwheat (20%)	Sc	8.10 × 10^7^	<1.00 × 10^4^ LAB		1.30 × 10^8^	<1.00 × 10^4^ LAB
		Sc+Wc wt	4.40 × 10^7^	1.36 × 10^9^		5.00 × 10^7^	1.35 × 10^9^
		Sc+Wc mut	3.80 × 10^7^	1.28 × 10^9^		6.90 × 10^7^	1.13 × 10^9^

Viable cells quantified as CFU counts per g present in pilot dough containing (60–20%) or (40–40%) of maize and rice supplemented with 20% of chickpea, quinoa, or buckwheat after 16 h of fermentation with *Saccharomyces cerevisiae* Lievital alone (Sc), or in combination with *W. cibaria* BAL3C-5 (Wc wt) or *W. cibaria* BAL3C-5 C120T (Wc mut) are depicted. * The concentration of presumptive indigenous LAB in control non-inoculated dough samples was determined by CFU counts on plates containing MRS supplemented with chloramphenicol (10 mg/L).

## Data Availability

Data is contained within the article.
